# Factors Influencing Pain Management of Patients with Osteoarthritis: A Cross-Sectional Study

**DOI:** 10.3390/jcm11051352

**Published:** 2022-03-01

**Authors:** Gyöngyi Anna Mezey, Zsuzsanna Máté, Edit Paulik

**Affiliations:** Department of Public Health, Albert Szent-Györgyi Medical School, University of Szeged, 6720 Szeged, Hungary; mate.zsuzsanna@med.u-szeged.hu (Z.M.); paulik.edit@med.u-szeged.hu (E.P.)

**Keywords:** self-medication, knee osteoarthritis, hip osteoarthritis, WOMAC

## Abstract

Background: Osteoarthritis (OA) is a complex disease associated with chronic pain. Many patients treat their joint pain at a symptomatic level with over-the-counter (OTC) pain medications, often without the knowledge of their physicians. The aim of this study was to provide physicians with data about osteoarthritic patients’ habits of pain management and to examine the explanatory factors of various ways of self-treatment. Methods: A cross-sectional study involving 189 patients with hip or knee OA and scheduled for joint replacement surgery was carried out. Participants filled out a self-administered questionnaire consisting of the Western Ontario and McMaster Universities Osteoarthritis Index and questions about their methods of alleviating pain. Results: 2.6% of patients did not use anything to alleviate their pain, while 63% practiced a non-pharmacological method. Diclofenac was the most frequently used drug, followed by ibuprofen. Profession had the greatest impact on medication habits; patients doing manual work were significantly more likely to take OTC non-steroidal anti-inflammatory drugs and use topical analgesics. Conclusions: Patients utilized a wide variety of pain management techniques. They seemed to use well-known painkillers, even if their side effects were less desirable. Such patients require comprehensive pain management, including educational and behavioural interventions, complemented by topical and oral medication.

## 1. Introduction

Osteoarthritis (OA) is a complex disease defined by the American College of Rheumatology (ACR) as “A heterogeneous group of conditions that lead to joint symptoms and signs which are associated with defective integrity of articular cartilage” [1]. Clinically, OA is characterized by joint pain, joint stiffness, gait abnormalities, and variable degrees of functional impairment [2]. Patients with chronic pain associated with musculoskeletal disorders have some of the poorest health-related quality of life (HRQoL) ahead of neurological, renal, and cardiovascular diseases, with severe restrictions in their work and ordinary activities of daily living [3].

As stated in the Global Health Estimates 2000–2019 study, OA was the 17th leading cause of total years lived with disability (YLD), with 1.8% of YLDs in 2000, but by 2019, it had become the 13th leading cause of YLDs at a global level with 2.3% of YLDs [4]; becoming the third-most rapidly rising condition associated with disability behind diabetes and dementia. Global prevalence of hip and knee OA is approaching 5% [5], and by 2030, it is predicted to reach 30% [6]. It is expected to become a major healthcare concern as the population ages, obesity rates rise, and more people adopt the Western lifestyle [7].

OA remains a relatively unaddressed public health concern compared to such chronic diseases as cancer, diabetes, and heart disease. There is a certain level of complacency and a fallacy that joint pains are an inevitable part of aging. The degradation of articular cartilage is often seen as a condition to be tolerated, not managed [8]. As many people do not recognise their joint pain as a disease, they often do not consult a health professional for its management. They treat their joint pain at a symptomatic level with over-the-counter (OTC) pain medications, similarly to a headache or lower back pain. A serious problem is that this practice is contrary to major clinical guidelines, which generally agree on a combination of non-pharmacological and pharmacological therapies. The ACR deems non-pharmacological therapies as the “cornerstone of OA management”, which should be maintained throughout the course of the disease, and stresses that pharmacological therapies should function as add-on therapy to non-pharmacological treatment [9]. The European League Against Rheumatism recommends the use of topical non-steroidal anti-inflammatory drugs (NSAIDs) and capsaicin as alternatives to oral analgesics or in combination with them [10]. 

Regarding oral analgesics, paracetamol was favoured previously by major clinical guidelines, recommending it to be the first choice in managing mild-to-moderate OA-related pain [9,11,12,13,14], while recently both NSAIDs and paracetamol are considered to be appropriate for treatment, though now a more conservative dosing for the latter is advised due to the increased risk of adverse events [15]. Beyond their burden on the cardiovascular system, it is widely accepted that regular use of NSAIDs increases the risk of interstitial nephritis, atrial fibrillation, and severe GI complications, including ulceration, bleeding, and perforation, by 2 to 4 times [16]. This fact is further aggravated by the increasing number of people who are opting for self-medication with OTC medications, without consulting a doctor, often under the influence of advertisements. Major and Vincze contacted 4536 specialists (pharmacists, pharmaceutical assistants, and pharmacy managers) to investigate the Hungarian patients’ habit of buying OTC medications. Their results show that 58.2% of patients buying OTC medications in pharmacies are usually self-reliant in self-medication, but they have little knowledge about these drugs [17]. 

The combination of OA patients’ needs for analgesics, the risk of concomitant use of multiple NSAIDs, and patients’ tendency for self-medication practices emphasizes the need for healthcare professionals to understand osteoarthritic patients’ health behaviour. Unfortunately, many times the attending physicians have no detailed data on what their patients use to alleviate their pain and in what quantities do they take OTC painkillers, a problem we wish to solve.

The aim of this study was to investigate osteoarthritic patients’ habits of pain management (both pharmacological and non-pharmacological) and to examine the explanatory factors of various ways of treatment.

## 2. Materials and Methods

### 2.1. Study Design and Participants

A cross-sectional study was performed by the Department of Public Health, University of Szeged, based on data collected at the Department of Orthopaedics, Albert Szent-Györgyi Health Care Centre, University of Szeged (Szeged, Hungary) and at the Orthopaedic Ward of Réthy Pál Hospital of Békés County Central Hospital (Békéscsaba, Hungary) from August 2019 to September 2020.

### 2.2. Inclusion and Exclusion Criteria

Only patients awaiting total knee or hip surgery were included, while patients receiving unicondylar knee arthroplasty were excluded. No other exclusion criteria were set, and participation was offered for all eligible patients in order to have the full list of eligible subjects, with every subject standing an equal chance of being included to reduce selection bias.

### 2.3. Data Collection

Data collection was carried out with the use of a paper and pencil questionnaire 24 h prior to surgery. Patients with knee or hip OA scheduled for joint replacement surgery were involved on a voluntary basis. Patients filled out the self-administered questionnaire in their rooms after receiving a detailed briefing and signing the informed consent form.

### 2.4. Variables

The questionnaire comprised three basic sections: 1, pain management; 2, measures of pain and functionality by WOMAC; 3, sociodemographic data (age, gender, etc.).

#### 2.4.1. Dependent Variables

To investigate pain management, the name, dose, and frequency of use of OTC and prescription-only medications were recorded. We only took into account regular medication use, that is, painkillers that participants used at least once a week. Based on these data, the following categories were made: total painkiller use (regular use of any type of painkiller), regular use of OTC oral NSAID, regular use of topical NSAID (cream/gel/patch), regular use of oral prescription medication, regular use of per os opioid-containing medication, and regular use of non-pharmacological methods. The dose of a medication was considered high if it reached or passed the recommended daily intake, that is: ibuprofen > 1800 mg/day, naproxen ≥ 1000 mg/day, diclofenac ≥ 150 mg/day, aceclofenac ≥ 100 mg/day, niflumic acid ≥ 750 mg/day, meloxicam ≥ 15 mg/day, piroxicam ≥ 20 mg/day, and nimesulide ≥ 200 mg/day [18].

We also examined non-pharmacological pain management techniques (e.g., physiotherapy, therapeutic massage, cold wraps, etc.). In this context, “physiotherapy” describes manual therapy and exercise therapy guided by a professional physiotherapist in order to build muscle strength and improve range of motion, while “exercising” includes basic warm-up and stretching exercises carried out by the patients in their homes, typically once a day in the morning to ease the joint stiffness they acquired during sleep. “Massage” specifically refers to massages carried out by the patients themselves. The term “cold wraps” includes chilled gel packs as well as towels soaked in cold water.

#### 2.4.2. Independent Variables

Pain and functionality were measured by the validated Hungarian version of the disease-specific Western Ontario and McMaster Universities Osteoarthritis Index (WOMAC), which contains 24 items, covering 3 dimensions: pain (5 items) during walking, using stairs, in bed, sitting or lying, and standing upright; stiffness (2 items) after waking up and later in the day; and function (17 items) using stairs, rising from sitting, standing, bending, walking, getting in/out of a car, shopping, putting on/taking off socks, rising from bed, lying in bed, getting in/out of bathtub, sitting, getting on/off toilet, heavy domestic duties, and light domestic duties. All items were scored on a scale of 1–10, totalling 24–240, where higher scores indicated increased pain and decreased function [19,20]. 

The WOMAC questionnaire was chosen for this study for its good reported internal consistency, excellent test–retest reliability, and experts’ involvement in development [21].

Regarding level of education, participants without a high school diploma were considered as ‘low’, with a high school diploma as ‘middle’, and with college and university diploma as ‘high’ level. Job profile was considered manual if the person’s job was physically demanding (e.g., manual labour), and non-manual if it was intellectual work (e.g., office environment). Body mass index (BMI) was calculated as weight in kilograms divided by height squared in meters. Based on the World Health Organization recommendations, patients with BMIs below 18.5 kg/m^2^ were categorised as underweight, those with BMIs between 18.5 kg/m^2^ and 24.9 kg/m^2^ as normal, BMIs between 25.0 kg/m^2^ and 29.9 kg/m^2^ as overweight, and anyone with a BMI over 30.0 as obese [22]. 

### 2.5. Statistical Analysis

Data analysis was carried out with IBM SPSS (Statistical Package for the Social Sciences) version 27 (SPSS Inc., Chicago, IL, USA). Descriptive statistics including frequency, percentage, mean, median, standard deviation (SD), and interquartile range (IQR) were performed to describe the study sample. After normality testing, the only variable not following a normal distribution was age, as OA predominantly affects the older generation. Association between categorical data was evaluated with a Chi-square test, and with one-way ANOVA for continuous data. Multivariable binary logistic regression analyses using the forward stepwise method were applied to determine the explanatory factors for analgesic use. The independent variables entered into the model were: gender, age group, level of education, job profile, affected joint, BMI category, and WOMAC total score. *p*-values for covariates to be included in the model were set at 0.05. Odds ratios (OR) and 95% confidence intervals (95% CI) were calculated, and statistical significance was set at *p* < 0.05.

## 3. Results

### 3.1. Characteristics of Patients

The baseline characteristics of the patients (*n* = 189) are shown in Table 1. The median age was 68 years (IQR: 12 years), and the majority of patients were women (70.1%). Nearly half of them had hip (48.7%) OA, and more than half (57.1%) were obese.

### 3.2. Characteristics of Treatment

Of the patients, 6.9% used neither pharmacological nor non-pharmacological methods to alleviate their pain, not even occasionally.

#### 3.2.1. Pharmacological Pain Management

The active ingredients of oral pain medications and the number of participants taking them are represented in Table 2. Diclofenac was the most frequently used drug, followed by ibuprofen and tramadol. Medications with paracetamol and selective cyclooxygenase-2 (COX-2) blockers were taken by 9 patients (4.8%) and 1 (0.5%) patient, respectively. A total of 24 participants (12.7%) took 2 different types of oral analgesic, while 9 (4.8%) and 2 patients (1.1%) took 3 or 4 different types, respectively. Also, 34 patients (18%) took high doses of painkillers regularly. Diclofenac was the most favoured with 15 patients reaching the recommended daily dose. A total of 38.1% of patients used prescription medication regularly.

#### 3.2.2. Non-Pharmacological Pain Management

The majority of patients (65.1%) practiced a non-pharmacological method to mitigate their pain, with 7.4% using these methods exclusively. Exercise, massages, and cold packs were the most favoured. A total of 29.1% used topical analgesics, all of which had diclofenac as an active ingredient, while 18.5% used a topical herbal cream with comfrey extract. Only 7 patients took part in physiotherapy, and of them, only 2 reported it as a regular (two times per week) activity (Table 3). 

While assessing the differences between knee and hip OA patients, knee patients were found to have significantly higher BMI (χ = 10.12, *p* < 0.01), but there were no other differences between the two groups’ demographic characteristics. Even though hip OA patients reported significantly worse HRQoL in the WOMAC total score (mean ± SD: 152.76 ± 41.23) than knee OA patients (139.62 ± 45.12) (*p* = 0.038), they were less likely to regularly use NSAIDs, either in tablet or cream form (42.2%), than were knee patients (59.8%), (χ^2^ = 5.72, *p* = 0.017).

Knee OA patients were significantly more likely to use topical analgesics (χ^2^ = 6.20, *p* = 0.013) and had a greater tendency to use non-pharmacological methods of pain management. 

### 3.3. Assessment of Influencing Factors 

Table 4 demonstrates the associations between different treatment forms and patients’ characteristics.

Results of the stepwise logistic regression analysis showed that manual work had the greatest impact on medication habits; patients with physically demanding jobs were significantly more likely to take painkillers in general, OTC NSAIDs, and topical analgesics. Degeneration of the knee joint specifically seems to be connected to manual work, as such patients were similarly more likely to use painkillers in general and topical analgesics. As shown in Table 4, WOMAC Score results showed that patients with poorer physical function and/or higher pain level were more likely to take prescription medications, each point increases the chance of taking medicine by 1.3.

All variables related to stress on the joint showed a greater likelihood of medication use except BMI, in the case of which, a higher value in fact suggested a bigger chance for topical analgesic use, but patients with higher BMIs were less likely to take OTC NSAIDs, compared to participants with normal BMIs. Regarding factors associated with non-pharmacological pain management, female patients showed a greater willingness to mitigate their pain in such ways (Table 5, Table 6, Table 7, Table 8 and Table 9).

## 4. Discussion

In this study, we aimed to investigate osteoarthritic patients’ habits of pain management and to examine the explanatory factors of various ways of self-treatment. OA is a disease for which pain is a main characteristic. Accordingly, patients in the current study utilized a wide variety of pain management technics. The majority of patients practiced a non-pharmacological method, with women in particular favouring it, while pharmacological methods were chosen by patients doing manual labour. Although more weight puts more strain on the joint, contrary to expectations, patients with higher BMI were less likely to take OTC NSAIDs.

A total of 23.3% of patients took OTC NSAIDs regularly. This is in line with the results (26.5%) from the 2011 Five European Countries National Health and Wellness Survey, as reported by Kingsbury et al. Our results for prescription medication use (38.1%) and paracetamol (1.0%) were comfortably within the range of the survey’s result of 33.0–53.2% and 0–6.0%, respectively. On the other hand, we experienced two major differences. On average, the use of opioid medications and COX-2 inhibitors was higher in the participating countries (France, Germany, Italy, Spain, and the UK) with 35.6% and 6.6%, compared to our results, 6.9% and 0.5%, respectively [23]. This infrequent use of paracetamol can be seen throughout Europe, barring the Nordic countries, where it is highly favoured [24]. Results showing that diclofenac and ibuprofen were the most-used active ingredients by our sample also correspond with results from European and Asian countries [23,24,25]. A total of 38.1% of patients used prescription medication; we can only hope that this fraction of the patient population was under the care of a specialist. It is, however, obviously important to assess also those who take NSAIDs on their own accord since the lack of professional supervision increases the chance of the complications that arise from self-medication, either by drug abuse or lack of mucosa protection. Medicines preferred by patients are a cause for concern, with diclofenac being the most popular but COX-2 inhibitors being neglected, as a study by Massó González et al. showed that the relative risk of upper GI bleeding/perforation was 4.50 for traditional NSAIDs, and 3.98 for diclofenac specifically, but only 1.88 for coxibs [26].

The distribution of active ingredients indicates that patients tend to use well-known pain medications even if their side effect profiles are less desirable. But questions arise even in case of professionally recommended medications, as some guidelines warn against the use of the previously favoured paracetamol, recommending it only conditionally, and stressing the importance of personalised therapies [27]. Knee OA patients were significantly more likely to use topical analgesics, which on one hand can be attributed to the fact that the knee joint is easier to reach and the active ingredients absorbed through the skin reach the site of pain more efficiently, but also knee OA patients had significantly higher BMIs compared to hip OA patients, and as the influencing factor assessment showed, patients with higher BMIs were over four times more likely to use topical analgesics compared to those with normal BMIs. Since hip OA patient with higher BMIs did not use more topical painkillers compared to those with lower BMIs, it seems that the fact that the knee joint is affected contributes more to the use of topical painkillers than BMI. The fact that only 29.1% of patients used topical analgesics is also a possible indicator that many patients managed their pain by themselves, even though professional guidelines (e.g., that of the ACR) favour topical drugs over oral medication as a way to decrease harmful GI side-effects [28]. It would be worth paying particular attention to hip OA patients who seem to prefer oral painkillers.

The importance of using topical analgesics cannot be overemphasised, given the advanced age of our patients, the high risk of co-morbidities, and the additional drug use associated with these conditions involving the risk of potential drug interactions. Beyond the well-known GI side effects caused by NSAIDs, with diclofenac being the most favoured OTC painkiller, the possibility of adverse cardiovascular events must not be overlooked. Schmidt et al. found that people taking diclofenac had a 20% increased rate for a major adverse cardiovascular event, such as a myocardial infarction, compared to patients taking paracetamol, and 30% compared to those who took naproxen [29].

Our study also showed high prevalence of the use of non-pharmacological techniques, which is fortunate, although typically limited to herbal creams, cold compressions, and in one case, warm baths, as Hungary has a long history of balneotherapy [30]. Although pain management methods that could be carried out by the patients themselves seem to be popular, unfortunately the prevalence of professionally guided physiotherapy was low compared to other studies [25,31]. Although 31.2% of patients reported doing exercise, it was practiced as a way of weight management. As 57.1% of patients were overweight and BMI was identified in this study as a risk factor, we wish to emphasise the systematic integration of weight management into the OA therapy course because obesity is suggested to be the main modifiable risk factor of OA [32]. A comprehensive and individualised plan for management of OA should therefore include educational, behavioural, psychosocial, and physical interventions, as well as topical, oral, and intraarticular medications [23]. We also wish to highlight the importance of preoperative physiotherapy. Unfortunately, patients are not routinely referred to physiotherapy within a year before surgery, even though studies showed that among patients who received preoperative physiotherapy a significant improvement was found for active and passive rotation, pain, daily functioning, vitality, psychological health, and social life [33].

For both hip and knee OA, the core treatments are exercise, education, mechanical interventions, and weight loss [34]. Given how few of our patients do exercise or receive physiotherapy, the question arises as to how much information patients have about non-pharmacological therapies. Because of this, we would like to encourage both general practitioners and specialists to recommend the following techniques to their patients taking their current condition into account. Manual therapy [35], transcutaneous electrical nerve stimulation, [36] and knee braces [37] are proven to reduce pain, the latter having the additional benefit of reducing knee instability, and they can also be effective when there is a valgus or varus deformity. To compensate for decreased muscular strength, resistance [38] and neuromuscular exercise [39] have been shown to be effective. Specifically for patients with hip OA, Nordic walking was found to build muscle strength and has been shown to be effective for weight loss, thus providing further benefits for OA patients [40,41].

In order to help patients maintain their exercise programmes, group-mediated cognitive behavioural physical activity intervention is advised [42].

By postponing non-urgent elective surgery during the COVID-19 pandemic, waiting times increased and the number of operations decreased. Compared to 2019, the median number of days on the waiting list before surgery increased by 88 days for knee replacements and by 58 days for hip replacements in 2020 [43]. In this situation, a further increase in self-medication by patients can be expected; thus, the responsibility of general practitioners in pain management has increased significantly. It is highly important that physicians are up to date on their new OA patients’ pain management habits so as to monitor habitual painkiller use and for their long-time patients to keep them updated with the current guidelines. The National Health Service Greater Glasgow and Clyde’s Guidelines for the management of chronic non-malignant pain intends to assist healthcare professionals in the choice of disease-specific treatments focusing on supporting self-management through five steps: Initial Assessment, Formulating a Pain Management Plan, Self-Management Strategies, Pharmacological Management Strategies, and Follow-up and Annual Referral if Indicated [44].

As data collection was carried out by self-administered questionnaires, inaccuracies in patients’ memories have the potential to distort our data. Although this study was carried out in two different health centres, findings may not be generalizable to the overall population since the population was not very large, only representing part of the country, and only consisting of severe OA patients. The limitations of our study are consistent with the nature of observational studies and the bias on patient selection, for which we tried to correct by selecting a large number of participants from two different counties of the country and enrolling consecutive patients. Also, the potential adverse effect of the NSAID use could not be determined because of a lack of data on such factors as antacid use. We wish to expand on this current study in the future by gathering information regarding the use of antacids to support risk assessment, and also the effect a successful replacement surgery has on the amount of medication patients take.

## 5. Conclusions

The use of NSAIDs in OA treatment, either planned by a medical professional or taken of the patients’ own accord, is very high. Two-thirds of the population affected by OA are over 65 (which is the standard age of retirement in Hungary), which carries the risk of comorbidities and the parallel use of several medications, but a considerable fraction of OA patients is still active, for whom immediate and long-lasting pain management is both medically and financially important. Both general practitioners and specialists need to familiarise themselves with their patients’ pain management habits and make a comprehensive and personalized plan for the management of OA patients.

## Figures and Tables

**Table 1:** Baseline characteristics of patients.

Characteristics	*n* (%)
Gender	
Men	56 (29.6)
Women	133 (70.4)
Age groups	
<65 years	65 (34.4)
≥65 years	124 (65.6)
Level of education	
Lower	82 (43.4)
Middle	69 (36.5)
Higher	38 (20.1)
Job profile	
Manual	97 (51.3)
Non-manual	92 (48.7)
Affected joint	
Hip	92 (48.7)
Knee	97 (51.3)
BMI categories (kg/m^2^)	
18.5–24.9	28 (14.8)
25.0–29.9	53 (28.1)
≥30.0	108 (57.1)
WOMAC Index	mean ± SD
Pain	29.23 ± 11.00
Stiffness	11.96 ± 4.47
Physical function	104.83 ± 31.91
Total score	146.02 ± 43.65

**Table 2:** Active ingredients of oral medications.

Medication	*n* (%)
NSAIDs	
Diclofenac	74 (39.2)
Ibuprofen	42 (22.2)
Aceclofenac	12 (6.3)
Acemetacin	10 (5.3)
Naproxen	9 (4.8)
Nimesulide	4 (2.1)
Piroxicam	3 (1.6)
Aspirin	3 (1.6)
Meloxicam	1 (0.5)
Niflumic acid	1 (0.5)
Lornoxicam	1 (0.5)
Coxibs	
Etoricoxib	1 (0.5)
Opioids	
Tramadol	13 (6.9)
Combinations	
Tramadol + paracetamol	6 (3.2)
Tramadol + dexketoprofen	1 (0.5)
Paracetamol + codeine phosphate	1 (0.5)
Paracetamol	2 (1.0)

**Table 3:** The occurrence of different treatment forms.

Form of Treatment	*n* (%)
Total painkiller use	95 (50.3)
Regular OTC oral NSAID	44 (23.3)
Topical NSAID cream/gel/patch	55 (29.1)
Prescription medication	72 (38.1)
Per os opioid-containing medication	18 (9.5)
Topical herbal cream	35 (18.5)
Non-pharmacological methods	124 (65.6)
Exercising	59 (31.2)
Massage	55 (29.1)
Cold packs	49 (25.9)
Warm bath	20 (10.6)
Physiotherapy	7 (3.7)
Kinesio tape	6 (3.2)
Magnetic band/patch	2 (1.1)

OTC: over the counter, NSAID: non-steroidal anti-inflammatory drug.

**Table 4:** Associations between different treatment forms and patients’ characteristics.

	All Painkillers *n* (%)	OTC NSAIDs *n* (%)	Prescription Painkillers *n* (%)	Topical NSAIDs *n* (%)	Opioid *n* (%)	Non-Pharma *n* (%)
Gender ^a^	*p* = 0.186	*p* = 0.443	*p* = 0.662	*p* = 0.649	*p* = 0.070	*p* < 0.001
Men	24 (42.9)	11 (19.6)	20 (35.7)	15 (26.8)	2 (3.6)	27 (48.2)
Women	71 (53.4)	33 (24.8)	52 (39.1)	40 (30.1)	16 (12.0)	97 (72.9)
Age group ^a^	*p* = 0.609	*p* = 0.256	*p* = 0.181	*p* = 0.098	*p* = 0.673	*p* = 0.160
<65 years	31 (47.7)	12 (18.5)	29 (44.6)	51 (78.5)	7 (10.8)	47 (72.3)
≥65 years	64 (51.6)	32 (25.8)	43 (34.7)	83 (66.9)	11 (8.9)	77 (62.1)
Level of education ^a^	*p* = 0.368	*p* = 0.385	*p* = 0.213	*p* = 0.251	*p* = 0.949	*p* = 0.086
Low	46 (56.1)	23 (28.0)	37 (45.1)	29 (35.4)	8 (9.8)	47 (57.3)
Middle	32 (46.4)	13 (18.8)	22 (31.9)	17 (24.6)	6 (8.7)	48 (69.6)
High	17 (44.7)	8 (21.1)	13 (34.2)	9 (23.7)	4 (10.5)	29 (76.3)
Job profile ^a^	*p* = 0.016	*p* = 0.011	*p* = 0.225	*p* = 0.030	*p* = 0.171	*p* = 0.615
Manual	57 (58.8)	30 (30.9)	41 (42.3)	35 (36.1)	12 (12.4)	62 (63.9)
Non-manual	38 (41.3)	14 (15.2)	31 (33.7)	20 (21.7)	6 (6.5)	62 (67.4)
Affected joint ^a^	*p* = 0.007	*p* = 0.625	*p* = 0.558	*p* = 0.013	*p* = 0.539	*p* = 0.101
Hip	37 (40.2)	20 (21.7)	37 (40.2)	19 (20.7)	8 (8.2)	55 (59.8)
Knee	58 (59.8)	24 (24.7)	35 (36.1)	36 (37.1)	10 (10.9)	69 (71.1)
BMI categories ^a^	*p* = 0.955	*p* = 0.056	*p* = 0.854	*p* = 0.034	*p* = 0.280	*p* = 0.104
18.5–24.9 kg/m^2^	14 (51.9)	11 (40.7)	10 (35.7)	4 (14.8)	2 (7.4)	21 (75.0)
25.0–29.9 kg/m^2^	26 (49.1)	9 (17.0)	19 (35.8)	22 (41.5)	8 (15.1)	39 (73.6)
≥30.0 kg/m^2^	55 (51.4)	24 (22.4)	43 (39.8)	29 (27.1)	8 (7.5)	64 (59.3)
WOMAC total score ^b^	*p* = 0.531	*p* = 0.077	*p* < 0.001	*p* = 0.351	*p* = 0.041	*p* = 0.086
Users	148.00 ± 42.39	156.20 ± 44.17	160.72 ± 37.70	150.65 ± 36.69	166.00 ± 31.55	149.96 ± 43.39
Non-users	144.01 ± 45.03	142.92 ± 43.17	136.97 ± 44.74	144.11 ± 46.20	143.91 ± 44.28	138.49 ± 43.52

*p* values: ^a^ results of chi-square tests; ^b^ results of one-way ANOVA, comparing users of a given treatment with non-users. Non-pharma: non-pharmacological methods, BMI: body mass index, WOMAC: Western Ontario and McMaster Universities Osteoarthritis Index.

**Table 5:** Forward stepwise logistic regression analysis of factors associated with regular painkillers use (last step).

Variable	OR (95% CI)	*p* Value
Job profile (manual)	2.253 (1.231–4.121)	0.008
Affected joint (knee)	2.440 (1.334–4.464)	0.004

Variables not entered into the model: gender, age group, level of education, BMI, WOMAC Total Score. OR: odds ratio, 95% CI: confidence interval, 95%; BMI: body mass index.

**Table 6:** Forward stepwise logistic regression analysis of factors associated with regular use of OTC NSAIDs (last step).

Variable	OR (95% CI)	*p* Value
Job profile (manual)	2.637 (1.270–5.479)	0.009
BMI categories		
25.0–29.9 kg/m^2^	0.274 (0.093–0.806)	0.019
≥30.0 kg/m^2^	0.387 (0.154–0.971)	0.043

BMI reference category: 18.5–24.9 kg/m^2^. Variables not entered in the model: gender, age group, level of education, affected joint, WOMAC Total Score. OR: odds ratio, 95%; CI: confidence interval, 95%; OTC: over the counter; NSAID: non-steroidal anti-inflammatory drug; BMI: body mass index.

**Table 7:** Forward stepwise logistic regression analysis of factors associated with regular use of prescription medication (last step).

Variable	OR (95% CI)	*p* Value
WOMAC Total Score (continuous)	1.013 (1.006–1.021)	<0.001

Variables not entered into the model: gender, age group, level of education, affected joint, BMI. OR: odds ratio, 95%; CI: confidence interval, 95%; BMI: body mass index.

**Table 8:** Forward stepwise logistic regression analysis of factors associated with regular use of topical analgesic (last step).

Variable	OR (95% CI)	*p* Value
Job profile (manual)	2.346 (1.183–4.651)	0.015
Affected joint (knee)	2.870 (1.403–5.871)	0.004
BMI categories		
25.0–29.9 kg/m^2^	4.261 (1.232–14.746)	0.022
≥30.0 kg/m^2^	1.656 (0.503–5.447)	0.407

BMI reference category: 18.5–24.9 kg/m^2^. OR: odds ratio, 95%; CI: confidence interval, 95%; BMI: body mass index. Variables not entered in the model: gender, age group, level of education, WOMAC Total Score.

**Table 9:** Forward stepwise logistic regression analysis of factors associated with non-pharmacological pain management (last step).

Variable	OR (95% CI)	*p* Value
Gender (women)	2.894 (1.513–5.537)	0.001

OR: odds ratio, 95%; CI: confidence interval, 95%. Variables not entered in the model: age group, level of education, job profile, affected joint, BMI, WOMAC Total Score.

## Data Availability

Not applicable.
